# A phase II study of paclitaxel, weekly, 24-hour continuous infusion 5-fluorouracil, folinic acid and cisplatin in patients with advanced gastric cancer

**DOI:** 10.1054/bjoc.2000.1295

**Published:** 2000-07-24

**Authors:** C Kollmannsberger, D Quietzsch, C Haag, T Lingenfelser, M Schroeder, J T Hartmann, W Baronius, V Hempel, M Clemens, L Kanz, C Bokemeyer

**Affiliations:** Department of Hematology/Oncology, University of Tuebingen Medical Center, Tuebingen; Department of Internal Medicine II, Klinikum Chemnitz, Chemnitz; Department of Hematology/Oncology/Gastroenterology, University of Dresden, Dresden; Department of Gastroenterology, Horst-Schmidt-Kliniken, Wiesbaden; Department of Hematology/Oncology, St. Johannes-Hospital, Duisburg; Department of Internal Medicine, Krankenanstalt der Boromaeerinnen Trier, Germany

**Keywords:** gastric cancer, metastatic, chemotherapy, paclitaxel, continuous infusion

## Abstract

To evaluate the toxicity and efficacy of combination chemotherapy with paclitaxel, cisplatin and 24 h continuous infusion of 5-FU/folinic acid in patients (pts) with unresectable, locally advanced or metastatic gastric adenocarcinoma. Forty-five chemotherapy-naive pts (28 male and 17 female) with a median age of 60 years (range 35–74) were enrolled. 5-FU 2 g/m^2^was given weekly over 24 h i.v. preceded by folinic acid 500 mg/m^2^as a 2 h infusion. Paclitaxel 175 mg/m^2^was administered as a 3 h-infusion on days 1 and 22 and cisplatin 50 mg/m^2^as 1 h infusion on days 8 and 29. Six weeks of therapy (days 1, 8, 15, 22, 29, 36) followed by 2 weeks rest were considered one cycle. A median of 3 cycles (range 1–4) were administered to 45 pts assessable for response, survival and toxicity. Five pts (11%) obtained a CR and 18 pts (40%) a PR (ORR 51%; 95% Cl: 35.8–66.3%). Responses were achieved in the liver, lymph nodes, lungs and at the site of the primary tumour. Nine pts (20%) had stable disease. Thirteen pts (29%) were considered to have failed treatment, 8 pts (18%) due to progressive disease and 5 pts (11%) who did not receive one complete cycle of therapy due to acute non-haematologic toxicity. The median progression-free and overall survival times were 9 months (range 1–36+) and 14 months (range 2–36+), respectively. Neutropenia WHO III°/IV° occurred in 7 pts (15%) with only 1 pt having grade IV. Additional non-haematologic WHO III°/IV° toxicities included nausea/vomiting in 5 (11%), alopecia in 22 (49%), and diarrhoea in 1 patient each (2%). Dose reductions or treatment delays were necessary in 8 pts (17%), mainly due to neutropenia. All pts were treated on an outpatient basis. The combination of paclitaxel, cisplatin and continuously infused 5-FU/folinic acid appears to be a highly active regimen for the treatment of pts with advanced gastric cancer. While the overall acceptable toxicity allows its use in the palliative setting, it may also be an attractive option to be tested for neoadjuvant or adjuvant treatment. © 2000 Cancer Research Campaign


*British Journal of Cancer* (2000) **83**(4), 458–462
© 2000 Cancer Research Campaign
doi: 10.1054/ bjoc.2000.1295, available online at http://www.idealibrary.com on IDEAL®

### A phase II study of paclitaxel, weekly, 24-hour continuous infusion 5-fluorouracil, folinic acid and cisplatin in patients with advanced gastric cancer

**C Kollmannsberger[1], D Quietzsch[2], C Haag[3], T Lingenfelser[4], M Schroeder[5], JT Hartmann[1], W Baronius[2], V Hempel[2], M Clemens[6], L Kanz[1] and C Bokemeyer[1]**

[1]Department of Hematology/Oncology, University of Tuebingen Medical Center, Tuebingen; [2]Department of Internal Medicine II, Klinikum Chemnitz, Chemnitz; [3]Department of Hematology/Oncology/Gastroenterology, University of Dresden, Dresden; [4]Department of Gastroenterology, Horst-Schmidt-Kliniken, Wiesbaden; [5]Department of Hematology/Oncology, St. Johannes-Hospital, Duisburg; [6]Department of Internal Medicine, Krankenanstalt der Boromaeerinnen Trier; Germany

**Summary** To evaluate the toxicity and efficacy of combination chemotherapy with paclitaxel, cisplatin and 24 h continuous infusion of 5-FU/folinic acid in patients (pts) with unresectable, locally advanced or metastatic gastric adenocarcinoma. Forty-five chemotherapy-naive pts (28 male and 17 female) with a median age of 60 years (range 35–74) were enrolled. 5-FU 2 g/m$^2$ was given weekly over 24 h i.v. preceded by folinic acid 500 mg/m$^2$ as a 2 h infusion. Paclitaxel 175 mg/m$^2$ was administered as a 3 h-infusion on days 1 and 22 and cisplatin 50 mg/m$^2$ as 1 h infusion on days 8 and 29. Six weeks of therapy (days 1, 8, 15, 22, 29, 36) followed by 2 weeks rest were considered one cycle. A median of 3 cycles (range 1–4) were administered to 45 pts assessable for response, survival and toxicity. Five pts (11%) obtained a CR and 18 pts (40%) a PR (ORR 51%; 95% Cl: 35.8–66.3%). Responses were achieved in the liver, lymph nodes, lungs and at the site of the primary tumour. Nine pts (20%) had stable disease. Thirteen pts (29%) were considered to have failed treatment, 8 pts (18%) due to progressive disease and 5 pts (11%) who did not receive one complete cycle of therapy due to acute non-haematologic toxicity. The median progression-free and overall survival times were 9 months (range 1–36+) and 14 months (range 2–36+), respectively. Neutropenia WHO III°/IV° occurred in 7 pts (15%) with only 1 pt having grade IV. Additional non-haematologic WHO III°/IV° toxicities included nausea/vomiting in 5 (11%), alopecia in 22 (49%), and diarrhoea in 1 patient each (2%). Dose reductions or treatment delays were necessary in 8 pts (17%), mainly due to neutropenia. All pts were treated on an outpatient basis. The combination of paclitaxel, cisplatin and continuously infused 5-FU/folinic acid appears to be a highly active regimen for the treatment of pts with advanced gastric cancer. While the overall acceptable toxicity allows its use in the palliative setting, it may also be an attractive option to be tested for neoadjuvant or adjuvant treatment. © 2000 Cancer Research Campaign

**Keywords** gastric cancer; metastatic; chemotherapy; paclitaxel; continuous infusion

Despite a declining incidence during the past 60 years, gastric cancer is still among the most common cancers in Europe (World Health Organization, 1998). Metastatic gastric cancer remains an incurable disease with a median survival time of only 4–8 months.

Randomized studies have demonstrated a survival benefit and impact on quality of life for patients with irresectable or metastatic gastric cancer treated with chemotherapy plus supportive care as compared to best supportive care alone (Pyrhönen et al, 1995; Glimelius et al, 1997). Thus, chemotherapy for advanced gastric cancer is now widely accepted in Europe and the US. A number of drugs have demonstrated activity such as 5-fluorouracil (5-FU), etoposide, doxorubicin, methotrexate and cisplatin. Combination regimens, usually based on 5-FU, have achieved response rates between 20 and 40% (Schipper and Wagener, 1996; Wils, 1996). Several studies have shown that the combination of 5-FU and cisplatin is active and well tolerated in patients with gastric cancer (Okada et al, 1991; Kim et al, 1993; Rougier et al, 1994; Wilke et al, 1996a).

*Received 25 January 2000*
*Revised 17 April 2000*
*Accepted 25 April 2000*

*Correspondence to:* C. Bokemeyer

Paclitaxel is an antimitotic agent with the unique cytotoxic mechanism of tubulin stabilization and polymerization, resulting in nonfunctional microtubules (Rowinsky et al, 1990). Paclitaxel exhibits an antitumour activity against various tumours including gastric cancer cell lines (Vanhoefer et al, 1995; Chang et al, 1996). Ajani et al obtained a 17% response rate using paclitaxel as a single agent in gastric cancer, with a tendency towards a higher response rate in patients receiving paclitaxel as continuous infusion over 24 hours. A 22% response rate was observed in pretreated patients with gastric cancer by Cascinu et al, with paclitaxel as single agent administered over 3 hours (Ajani et al, 1998; Cascinu et al, 1998). Studies investigating the combination of paclitaxel with 5-FU revealed response rates between 15 and 50%, associated with a rather low toxicity profile (Cascinu et al, 1997; Murad, 1999). We have reported the results for the combination of 5-FU given as a weekly 24-hour continuous infusion plus folinic acid for 6 consecutive weeks in combination with paclitaxel at 3 weekly intervals (Bokemeyer et al, 1997a). A response rate of 32% was observed in 22 chemonaive gastric cancer patients resulting in a progression-free and overall survival of 8 and 11 months, respectively. Toxicity was mild with neutropenia (14%), alopecia (45%) and nausea and vomiting (5%) being the most frequent WHO grade III/IV toxicities (Bokemeyer et al, 1997a).

Two recent studies examined the combination of paclitaxel, cisplatin and 5-FU in patients with locally advanced adenocarcinoma of the oesophagus in the neoadjuvant setting, as well as in patients with metastatic disease. A high response rate combined with an acceptable toxicity was reported (Belani et al, 1997; Ilson et al, 1998).

Based on the activity of 5-FU and cisplatin in gastric cancer and on the favourable results of the combination of 5-FU/folinic acid and paclitaxel, we here present a phase II study combining weekly 24 h continuous infusion 5-FU/folinic acid with cisplatin and paclitaxel in an alternating schedule in patients with gastric cancer.

### PATIENTS AND METHODS

### Patients

Patients with histologically proven advanced or metastatic gastric cancer were included in this study. Inclusion criteria were as follows: unresectable, locally-advanced or metastatic disease; ECOG performance status 0–2; presence of measurable disease; age between 18 and 75 years; life expectancy > 3 months; no prior chemotherapy; adequate haematological, renal and hepatic function as defined by a granulocyte count ≥ $1.5 \times 10^9$/l, thrombocytes ≥ $100 \times 10^9$/1, serum creatinine ≤ 1.5 mg/dl, creatinine clearance ≥ 60 ml/min, bilirubin level < 2-fold and liver enzymes < 3-fold the upper normal limits and written informed consent. All patients had to be available for follow-up.

Patients were excluded from the study in case of one of the following: active bleeding, diffuse, non-measurable liver metastases; history of a secondary malignancy except for non-melanomatous skin cancer or carcinoma in situ of the cervix; concurrent insufficiently treated disease such as heart, renal or hepatic failure or uncontrolled infection; prior chemotherapy; presence of a concurrent psychiatric disorder; pregnancy.

Prior to therapy, a clinical history and physical examination, a complete blood count and serum chemistry including liver and kidney function tests, a creatinine clearance as well as an electrocardiogram and an audiogram were obtained. All measurable tumour lesions were radiologically assessed either by CT-scan, chest-X-ray or abdominal ultrasound.

The study was approved by the Ethics Committee of the University of Tuebingen.

### Treatment plan

Treatment was given once weekly for a total of 6 weeks followed by 2 weeks rest. This period was defined as one treatment cycle (Figure 1). 5-Fluorouracil was administered weekly at a dose of 2000 mg/m$^2$ as 24 h-continuous infusion preceded by 500 mg/m$^2$ folic acid as a 2 h infusion. Paclitaxel was administered at a dose of 175 mg/m$^2$ on days 1 and 22 as a 3 h infusion. All patients received dexamethasone 20 mg, ranitidine 300 mg as well as diphenhydramine 50 mg 30 minutes prior to paclitaxel in order to avoid hypersensitivity reactions. Cisplatin 50 mg/m$^2$ was added on days 8 and 29 to the 24 h-infusion 5-FU backbone of the regimen. All patients received adequate antiemetic pre-medication prior to chemotherapy. A permanent venous access (PORT-A-Cath, Baxter Inc Germany) was implanted in all patients in order to facilitate the continuous 5-FU application. Portable single use 24 h infusion pumps (Infusor LV 5®, Baxter Inc, Germany) were used for the ambulatory application of 5-FU. All patients were treated on an outpatient basis.

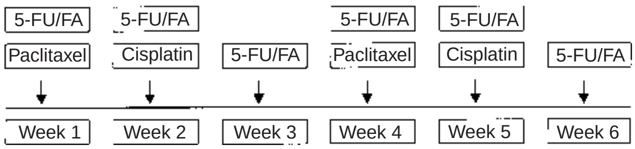

FA, folinic acid

Repeated on day 50

**Figure 1** Treatment plan

### Response and toxicity evaluation

Complete blood count, history and physical examination were recorded weekly. Renal and liver function tests were assessed biweekly in order to evaluate toxicity. Prior to each cycle, creatinine clearance, audiogram and electrocardiogram were rechecked. Toxicity was recorded every week based on WHO Toxicity Criteria. Dose modifications and treatment delays were performed as necessary according to the extent of the haematological and clinical toxicity. Planned dose modifications included the following: 25% dose reduction of paclitaxel and cisplatin in case of WHO grade 2 peripheral neurotoxicity; stop of paclitaxel and cisplatin in case of WHO grade 3/4 peripheral neurotoxicity; a 25% dose reduction of paclitaxel and/or 5-FU in case of WHO grade 3/4 granulocytopenia; 25% dose reduction of all 3 drugs in case of mucositis WHO grade ≥ 2. A 1-week treatment delay was planned in case of a WHO grade 3–4 toxicity, that had not resolved by the next week of treatment. Growth factors were not routinely used in the present study.

Baseline assessment of subjective symptoms (abdominal pain, vomiting, pain, loss of appetite, loss of weight) was performed with a modified EORTC-questionnaire by the patients themselves and their changes in intensity were recorded at the beginning, in the middle and at the end of each cycle. Patients were asked to grade their symptoms (absent, low, moderate, or severe) as well as their overall health status and quality of life on a scale reaching from 0 (very bad) to 100 (excellent). Questionaires were delivered by the treating physician and were to be completed prior to chemotherapy application.

Assessment of measurable disease was performed after each cycle according to WHO criteria (Miller et al, 1981). Complete remission (CR) was defined as the complete disappearance of all clinical, radiological and biochemical evidence of the disease and partial response as a greater than 50% reduction of all measurable tumour lesions lasting for at least 4 weeks. Stable disease was considered for all patients who achieved less than a partial response but no evidence of progressive disease (new lesions or increase in any area of measurable disease >25%). Treatment was continued if the patient showed a remission or stable disease. A maximum of 3 treatment cycles were planned per patient.

### Study endpoints

Objectives of the present study were the determination of the overall (complete and partial) response rate, the median progression-free survival (PFS), the overall survival (OS) and the toxicity of the treatment. PFS, follow-up duration and OS were calculated

*© 2000 Cancer Research Campaign*

**Table 1** Patient characteristics ($n = 45$)

| Characteristic | No. of patients (%) |
|---|---|
| Median age | 60 [35–74] |
| Male/female | 28 (62)/17 (38) |
| ECOG performance status (median) | 1 [0–2] |
| Gastric adenocarcinoma | 45 (100) |
| Prior surgery | |
| Gastrectomy | 14 (31) |
| Other | 2 (4) |
| Metastatic sites | |
| Tumour at the primary site[a] | 26 (58) |
| Lymph nodes | 31 (68) |
| Liver | 17 (38) |
| Peritoneum[a] | 15 (33) |
| Lungs | 5 (11) |
| Bone | 3 (7) |
| Other | 8 (18) |
| Median follow up | 14 months (1–36 months) |

[a]All patients with tumour at the primary site or peritoneal metastases had additional measurable lesions

**Table 2** Response and survival in 45 patients

| Response | No. of patients (%) |
|---|---|
| CR | 5 (11) |
| PR | 18 (40) |
| NC | 9 (20) |
| PD | 8 (18) |
| Early termination of treatment | 5 (11) |
| Survival | |
| Median overall survival | 14 months [2–36+ months] |
| Median progression-free survival | 9 months [1–36+ months] |

from the start of treatment to the date of disease progression or the date of the last evaluation or death, respectively. Survival curves were estimated by the method of Kaplan-Meier (Kaplan and Meier, 1958). All statistics was performed using SAS software (SAS Institute, Cary, NC, version 6.11). Quality of life questionaires were descriptively analysed.

### RESULTS

Forty-five patients with a median age of 60 years [35–75 years] were enrolled in the study. Patient characteristics are given in Table 1. Tumour at the primary site, lymph nodes and liver metastases as well as peritoneal metastases were the most common tumour sites and all patients had bidimensionally measurable tumour lesions. Fourteen patients had undergone gastrectomy prior to chemotherapy and 2 patients had received a gastroenterostomy.

### Response and survival

All patients were assessable for response and survival. Overall, 96 complete treatment cycles were administered to the 45 patients (median 3 cycles per patient; range 0–3 cycles). Five patients (11%) did not receive one complete treatment cycle for the following reasons: one patient refused further therapy due to WHO grade III° nausea and vomiting; one patient died of tumour bleeding following the second week of therapy; 3 patients were removed from therapy due to 5-FU related central neurotoxicity (1 patient), 5-FU-related cardiotoxicity with angina pectoris (1 patient) and a severe anaphylactic reaction to paclitaxel (1 patient). These patients were considered as treatment failures.

Twenty-three patients [51%; 95% CI: 35.8–66.3.0%] achieved an objective response including 5 complete remissions (11%). Nine patients (20%) had stable disease as the best response to therapy and the remaining 8 patients (18%) developed progressive disease during treatment. Taking only the 40 patients into account, who received at least one complete cycle of therapy, the overall response rate was 58% [95% CI: 40.9–73.0%]. Responses occurred mainly at the primary tumour site and at liver, lymph node and lung metastases. The first patient with a complete remission presented with tumour at the primary site, liver metastases, an adrenal metastasis as well as a soft tissue metastasis. Following palliative gastrectomy and 3 cycles of chemotherapy complete remission was proven by CT scan. In two patients a complete remission, as confirmed by CT scan, was obtained after 3 cycles of chemotherapy for tumour at the primary site and liver metastases. One of these patients had had a palliative partial gastrectomy prior to chemotherapy. Two further patients with metastatic disease to the liver and to the lymph nodes and peritoneum had undergone a gastrectomy prior to chemotherapy. Both subsequently received 3 cycles of chemotherapy and a complete remission was confirmed by CT-scan. Two of these 5 patients relapsed, both at their initial disease sites. The durations of complete responses are currently 8+, 13, 18+, 36 and 36+ months.

Interestingly, 7 of 15 assessable patients with, among others, peritoneal metastases achieved a remission in the peritoneum, two patients a complete and five patients a partial remission. All remission evaluations were based on CT scan (not confirmed by laparoscopy). After a median follow-up of 14 months [1–36+], 17 patients have died of their disease and twenty-three patients are alive with 6 of them having progressive disease. Seven patients received a second-line chemotherapy, 4 of them mitomycin C (Hartmann et al, 1999). The median progression-free and overall survival are 9 (range 1–36+ months; 95% CI: 7–11) and 14 months (range 2–36+; 95% CI: 7–21), respectively.

Quality of life questionnaires were obtained from 32 patients and revealed an improvement in symptoms and overall well being in 14 patients (44%) and a subjectively stable condition in 12 patients (38%). Only 6 patients (19%) complained about a worsening of symptoms and physical well-being during therapy. Improvement of symptoms and overall well-being was clearly associated with tumour response. Twenty patients with an objective response and 6 patients with stable disease reported an improvement or stable status following therapy. Progressive disease was accompanied by a deterioration of symptoms in 4 patients. Only 1 patient who had obtained a complete remission complained about a worsening of symptoms and 1 patient with progressive disease reported an improvement of symptoms.

### Toxicity

Toxicity according to WHO criteria was assessable in all 45 patients and is listed in Table 3 as the worst toxicity per patient during the total study period. Seven patients (15%) developed a WHO grade III/IV neutropenia. Based on the total number of cycles completed, neutropenia occurred in 17 of 96 cycles (18%). The duration of neutropenia was generally short and resolved

© 2000 Cancer Research Campaign

**Table 3** Toxicity (worst toxicity per patients during study (*n* = 45))

| Toxicity | WHO grade I/II No. patients (%) | WHO grade III/IV No. patients (%) |
|---|---|---|
| Neutropenia | 24 (53) | 7 (15) |
| Thrombocytopenia | 3 (7) | 0 |
| Fever/infection | 8 (18) | 1 (2) |
| Alopecia | 13 (29) | 27 (71) |
| Nausea/vomiting | 20 (44) | 5 (11) |
| Mucositis | 15 (33) | 2 (4) |
| Allergy | 0 | 1 (2)[b] |
| Neurotoxicity | 15 (33) | 1 (2)[a] |
| Diarrhoea | 11 (24) | 1 (2) |
| Hand-food syndrome | 5 (11) | 0 |
| Constipation | 9 (20) | 0 |
| Myalgia | 9 (20) | 0 |
| Nephrotoxicity | 10 (22) | 0 |
| Other | 0 | 1 (2) [a](cardiotox) |

[a]Probably 5-FU related.
[b]Flush, generalized urticaria and exanthema.

within 5 days. Only one patient had to be admitted to the hospital for WHO grade III fever and infection. This patient quickly responded to antibiotics without further complications, although the focus of the infection could not be detected. There was no WHO grade III°/IV° thrombocytopenia. The major WHO grade III/IV non-haematological toxicities were alopecia (71%), nausea/vomiting (11%) and mucositis (4%). No severe hand-foot syndrome was observed. In five patients treatment had to be stopped within the first cycle of therapy due to acute toxicities as stated above. Dose reductions (7 patients; 16%) and/or treatment delays (1 patient; 2%) were necessary in 8 patients (18%), in 7 patients due to neutropenia WHO grade III/IV and in 1 patient due to WHO grade III diarrhea. No dose reduction was performed due to neurotoxicity.

### DISCUSSION

Second generation protocols for the treatment of advanced gastric cancer are mainly based on 5-FU, high-dose MTX, cisplatin and antracyclines. In phase II trials, response rates up to 60% have been reported for regimens such as FAMTX, ELF, EAP, Cisplatin/5-FU or ECF (Klein et al, 1986; Preusser et al, 1989; Wilke et al, 1990; Findley and Cunningham, 1993; Wils, 1996). However, in randomised phase III trials, this high level of activity has only been confirmed for the ECF-regimen (Webb et al, 1997; Waters et al, 1999), whereas for the FAMTX, ELF or cisplatin/5-FU-regimen response rates of 20–25% have been reported (Kelsen et al, 1992; Wilke et al, 1995). In addition, particularly the FAMTX and EAP regimens were associated with severe toxicity. Thus, to date, ECF appears to be the most active regimen, but a definitive standard regimen for the palliative treatment of metastatic gastric cancer has not yet been defined.

Several new agents have been tested in gastric cancer in the past years including topoisomerase I inhibitors such as CPT-11 or taxanes such as paclitaxel. In vitro and in vivo data appear to support the use of paclitaxel in gastric cancer (Chang et al, 1996; Ajani et al, 1998; Cascinu et al, 1998). An additive cytotoxic effect has been reported in vitro for the sequence of paclitaxel followed by 5-FU whereas the exposure to 5-FU followed by paclitaxel showed subadditive effects (Kano et al, 1996). Based on these rationale we had previously performed a phase II trial examining the efficacy and toxicity of the combination of paclitaxel, 5-FU and folinic acid in 22 patients with chemonaive gastric cancer (Bokemeyer et al, 1997a). A response rate of 32% and a median overall survival of 11 months were achieved. Toxicity was low with neutropenia WHO grade III/IV occurring in 14% of patients. In the study presented here as well as in our previous study, 5-FU was administered as a protracted, weekly 24 h infusion combined with folinic acid, since this mode of application appears to be less toxic and potentially more active as compared to the bolus administration. This regimen was initially investigated in patients with colon cancer and showed a good activity with low toxicity (Ardalan et al, 1991). In gastric cancer, one study had reported the use of protracted 24 h continuous infusion of high-dose 5-FU achieving a remission rate of 24% and a stable disease rate of 59% in patients previously treated with bolus 5-FU therapy (Vanhoefer et al, 1994). Thus, the weekly administration of 24 h continuous infusion of high-dose 5-FU (2000 mg/m$^2$) preceded by folinic acid (500 mg/m$^2$ as 2-h infusion) has formed the backbone for the development of our treatment regimen. Paclitaxel was added at a standard-dose of 175 mg/m$^2$ on days 1 and 22.

The present study investigates the efficacy and toxicity of the same regimen of paclitaxel and weekly 24-hour continuous infusion 5-FU plus folinic acid, but with the addition of cisplatin. Cisplatin has been shown to be active in gastric cancer as a single agent as well as in combination with 5-FU (Rougier et al, 1994; Schipper and Wagener, 1996; Wilke et al, 1996b). Furthermore, a synergistic effect has been described not only for the combination of 5-FU and cisplatin (Schabel et al, 1979; Scanlon et al, 1986) but also for the combination of paclitaxel and cisplatin in human gastric cancer cell lines (Harstrick et al, 1994). In order to avoid intensive toxicity in palliatively treated patients, cisplatin was given in a dose of 50 mg/m$^2$ on days 8 and 29. An objective response rate of 51% – or 58% if only those patients receiving at least one complete cycle of therapy are taken into account – including 11% (13%) complete remissions in chemonaive gastric cancer patients demonstrates the high activity of this regimen. A similar favourable response rate of 51% with a median survival time of 6 months was recently achieved by Kim et al (1999) administering paclitaxel at a dose of 175 mg/m$^2$ on day 1, 5-FU at a dose of 750 mg/m$^2$ on days 1–5 as continuous infusion and cisplatin at a dose of 20 mg/m$^2$ as a 30 minute infusion on days 1–5. In patients with adenocarcinoma of the oesophagus, Ilson et al (1998) reported a response rate of 46% with a similar schedule. The median overall and progression-free survival of 14 and 9 months duration, respectively, in the current study are encouraging. The addition of cisplatin to our previous regimen consisting of paclitaxel and 5-FU/folinic acid appears to improve the overall response rate, in particular the number of complete responses and the median survival time (Bokemeyer et al, 1997b).

The toxicity of the present combination of paclitaxel, 24 h continuous infusion of high-dose 5-FU/folinic acid and cisplatin was acceptable and the treatment could be safely administered on an outpatient basis. Only one patient had to be hospitalized due to fever and infection. Dose reductions or treatment delays were performed in 18% of patients, mainly due to neutropenia. More than 60% of the patients who participated in the quality of life evaluation reported an improvement or stabilization of their symptoms and general condition. This clinical benefit not only reflects the favourable toxicity profile but also demonstrates the palliative effect of this regimen.

*© 2000 Cancer Research Campaign*

However, results of phase II trials have to be carefully interpreted since patient selection may cause a bias and subsequently misleading results. Thus, in order to confirm the high activity of the current regimen and in order to define the role of paclitaxel, randomized phase III trials are needed. The EORTC study 40953 has compared ELF to FAMTX and to 5-FU/folinic acid as well as to 5-FU/folinic acid plus cisplatin. Results of this study may yield a control arm for future randomized trials. Due to its high response rate and its moderate toxicity allowing outpatient treatment, the combination of high-dose 5-FU/folinic acid, cisplatin plus paclitaxel may be an attractive option to be tested in neoadjuvant or adjuvant trials in patients with locally advanced disease.

### REFERENCES

Ajani JA, Fairweather J, Dumas P, Patt YZ, Pazdur R, Mansfield PF (1998) Phase II study of Taxol in patients with advanced gastric carcinoma. *Cancer J Sci Am* **4**: 269–274

Ardalan B, Chua L, Tian EM, Reddy R, Sridhar K, Benedetto P, Richman S, Legaspi A, Waldman S, Morrell L et al (1991) A phase II study of weekly 24-hour infusion with high-dose fluorouracil with leucovorin in colorectal carcinoma [see comments]. *J Clin Oncol* **9**: 625–630

Belani C, Luketich JD, Landreaneau RJ, Kim R, Ramanathan RK, Day R, Ferson PF, Keean RJ, Posner M, Seeger J, Lembersky B (1997) Efficacy of cisplatin, 5-flurouracil, and paclitaxel regimen for carcinoma of the esophagus. *Semin Oncol* **24**: 89–92

Bokemeyer C, Lampe CS, Clemens MR, Hartmann JT, Quietzsch D, Forkmann L, Kollmannsberger C, Kanz L (1997a) A phase II trial of paclitaxel and weekly 24 h infusion of 5-fluorouracil/folinic acid in patients with advanced gastric cancer. *Anticancer Drugs* **8**: 396–399

Bokemeyer C, Hartmann JT, Lampe CS, Clemens MR, Quietzsch D, Forkmann L, Kanz L (1997b) Paclitaxel and weekly 24-hour infusion of 5-fluorouracil/folinic acid in advanced gastric cancer. *Semin Oncol* **24**: S19–S19

Cascinu S, Ficarelli R, Safi MA, Graziano F, Catalano G, Cellerino R (1997) A phase I study of paclitaxel and 5-fluorouracil in advanced gastric cancer. *Eur J Cancer* **33**: 1699–1702

Cascinu S, Graziano F, Cardarelli N, Marcellini M, Giordani P, Menichetti ET, Catalano G (1998) Phase II study of paclitaxel in pretreated advanced gastric cancer. *Anticancer Drugs* **9**: 307–310

Chang YF, Li LL, Wu CW, Liu TY, Lui WY, P'eng FK, Chi CW (1996) Paclitaxel-induced apoptosis in human gastric carcinoma cell lines. *Cancer* **77**: 14–18

Findley M, Cunningham D (1993) Chemotherapy of carcinoma of the stomach. *Cancer Treat Rev* **19**: 29–44

Glimelius B, Ekstrom K, Hoffman K, Graf W, Sjoden PO, Haglund U, Svensson C, Enander LK, Linne T, Sellstrom H, Heuman R (1997) Randomized comparison between chemotherapy plus best supportive care with best supportive care in advanced gastric cancer. *Ann Oncol* **8**: 163–168

Harstrick A, Vanhoefer U, Wilke H et al (1994) Interaction of taxol with cisplatin, etoposide and 5-FU in human gastric carcinoma cell lines. *Proc Am Assoc Cancer Res* **35**: 332

Hartmann JT, Quietzsch D, Daikeler T, Kollmannsberger C, Meyer F, Kanz L, Bokemeyer C (1999) Mitomycin C continuous infusion as salvage chemotherapy in pretreated patients with advanced gastric cancer. *Anti-Cancer Drugs* **10**: 729–733

Ilson DH, Ajani JA, Bhalla K, Forastiere AA, Huang Y, Patel P, Martin L, Donegan J, Pazdur R, Reed C, Kelsen DP (1998) Phase II trial of paclitaxel, fluororuacil, and cisplatin in patients with advanced carcinoma of the esophagus. *J Clin Oncol* **16**: 1826–1834

Kano Y, Akutsu M, Tsunoda S, Ando J, Matsui J, Suzuki K, Ikeda T, Inoue Y, Adachi KI (1996) Schedule-dependent interaction between paclitaxel and 5-fluorouracil in human carcinoma cell lines in vitro. *Br J Cancer* **74**: 704–710

Kaplan E, Meier P (1958) Nonparametric estimation from incomplete observations. *Am J Stat Assoc* **53**: 457–481

Kelsen DP, Atiq OT, Saltz L et al (1992) FAMTX versus etoposide, doxorubicin, and cisplatin: a random assignment trial in gastric cancer. *J Clin Oncol* **10**: 541–548

Kim NK, Park YS, Heo DS, Suh C, Kim SY, Park KC, Kang YK, Shin DB, Kim HT, Kim HJ et al (1993) A phase III randomized study of 5-fluorouracil and cisplatin versus 5-fluorouracil, doxorubicin, and mitomycin C versus 5-fluorouracil alone in the treatment of advanced gastric cancer. *Cancer* **71**: 3813–3818

Kim YH, Shin SW, Kim BS, Kim JH, Kim JG, Mok YJ, Kim CS, Rhyu HS, Hyun JH, Kim JS (1999) Paclitaxel, 5-fluorouracil, and cisplatin combination chemotherapy for the treatment of advanced gastric carcinoma. *Cancer* **85**: 295–301

Klein HO, Wickramanayak PD, Farrkh GR et al (1986) 5-fluroruracil (5-FU), adriamycin (ADM) and methotrexate (MTX) – a combination protocol (FAMTX) for treatment of metastasized stomach cancer. *Proc Am Soc Clin Oncol* **84**: 86

Miller AB, Hoodgstraten B, Staquet M, Winkler A (1981) Reporting results of cancer treatment. *Cancer* **47**: 207–211

Murad AM (1999) Chemotherapy for advanced gastric cancer: Focus on new agents and combinations. *Cancer Control* **6**: 361–368

Okada Y, Anai H, Hattori T, Maehara Y, Nishimura J, Sugimachi K, Nawata H (1991) Cisplatin plus continuous infusion of 5-fluorouracil for 5 days effective for patients with advanced gastric cancer. *Anticancer Drugs* **2**: 453–456

Preusser P, Wilke H, Achterrath W, Fink U, Lenaz L, Heinicke A, Meyer J, Meyer HJ, Buente H (1989) Phase II study with etoposide, doxorubicin, and cisplatin in advanced and measurable gastric cancer. *J Clin Oncol* **9**: 1310–1317

Pyrhönen S, Kuitunen Z, Nyandoto P et al (1995) Randomized comparison of fluorouracil, epidoxorubicin, and methotrexate (FEMTX) plus supportive care with supportive care alone in patients with non-resectable gastric cancer. *Br J Cancer* **71**: 587–591

Rougier P, Ducreux M, Mahjoubi M, Pignon JP, Bellefqih S, Oliveira J, Bognel C, Lasser Ph, Ychou M, Elias D, Cvitkovic E, Armand JP, Droz J-P (1994) Efficacy of combined 5-fluroruracil and cisplatin in advanced gastrc carcinomas. A phase II trial with prognostic factor analysis. *Eur J Cancer* **30A**: 1263–1269

Rowinsky EK, Cazenave LA, Donehower RC (1990) Taxol: a novel investigational antimicrotubule agent. *J Natl Cancer Inst* **82**: 1247–1259

Scanlon KJ, Newman EM, Lu Y, Priest DG (1986) Biochemical basis for cisplatin and 5-fluorouracil synergism in human ovarian carcinoma cells. *Proc Natl Acad Sci USA* **83**: 8923–8925

Schabel FM, Trader ML, Laster WR, Corbett TH, Griswold DP (1979) Cis-dichlorodiammine-platinum (II): combination chemotherapy and cross resistance studies with tumor of mice. *Cancer Treat Rep* **63**: 1459–1473

Schipper DL, Wagener DJ (1996) Chemotherapy of gastric cancer. *Anticancer Drugs* **7**: 137–149

Vanhoefer U, Wilke H, Weh J, Clemens M, Harstrick A, Stahl M, Hossfeld DK, Seeber S (1994) Weekly high-dose 5-FU and folinic acid as salvage treatment in advanced gastric cancer. *Ann Oncol* **5**: 850–851

Vanhoefer U, Harstrick A, Wilke H, Schleucher N, Walles H, Schroder J, Seeber S (1995) Schedule-dependent antagonism of paclitaxel and cisplatin in human gastric and ovarian carcinoma cell lines in vitro. *Eur J Cancer* **31A**: 92–97

Waters JS, Norman A, Cunningham D, Scarffe JH, Webb A, Harper P, Jofe JK, Mackean M, Mansi J, Leahy M, Hill A, Oates J, Rao S, Nicolson M, Hicksih T (1999) Long-term survival fater epirubicin, cisplatin and fluorouracil for gastric cancer: results of a randomized trial. *Br J Cancer* **80**: 269–272

Webb A, Cunningham D, Scarffe JH, Harper P, Norman A, Joffe JH, Hughes M, Mansi J, Findley M, Hill A, Oates J, Nicolson M, Hicksih T, O'Brian M, Iveson T, Watson M, Underhill C, Wardley A, Meehan M (1997) Randomized trial comparing epirubicin, cisplatin, and fluorouracil versus fluorouracil, doxorubicin, and methotrexate in advanced esophagogastric cancer. *J Clin Oncol* **15**: 261–167

Wilke H, Preusser P, Fink U, Achterrath W, Lenaz L, Stahl M, Schober C, Link H, Meyer HJ, Lucke B et al (1990) High-dose folinic acid/etoposide/5-fluroruracil in advanced gastric cancer-a phase II study in elderly patients or patients with cardiac risk. *Invest New Drugs* **8**: 65–70

Wilke H, Wils J, Rougier P et al (1995) Preliminary analysis of a randomized phase III trial of FAMTX versus ELF versus cisplatin/5-FU in advanced gastric cancer (GC). Atrial of the EORTC Gastrointestinal Tract Cooperative Group and the AIO (Arbeitsgemeinschaft Internistische Onkologie). *Proc Am Soc Clin Oncol* **14**: 206

Wilke H, Korn M, Vanhofer U, Fink U, Stahl M, Preusser P, Kohne C, Klassen U, Harstrick A, Schmoll HJ, Seeber S (1996a) Weekly infusional 5-fluorouracil plus/minus other drugs for the treatment of advanced gastric cancer. *J Infus Chemother* **6**: 123–126

Wilke H, Korn M, Köhne C, Fink U, Preusser P, Vanhoefer U, Wiegmann T, Stahl M, Harstrick A, Klassen U, Schmoll HJ, Seeber S (1996b) Phase II results of weekly infusional high-dose FU (HD-FU) plus folinic caid (FA) and biweekly cisplatin (C) in advanced gastric cancer. *Ann Oncol* **7(suppl 5)**: 46

Wils J (1996) The treatment of advanced gastric cancer. *Semin Oncol* **23**: 397–406

World Health Organization (1998) World Health Report 1998: A vision for all. World Health Organization, Geneva, Switzerlan

© 2000 Cancer Research Campaign
